# Wounds of the Heart

**Published:** 1853-08

**Authors:** G. J. Guthrie


*Wounds of the Heart.* From Lectures by G. J. Guthrie, Esq., F. R. S.—Wounds of the Heart are for the most part immediately fatal. Many persons have, however, been known to live for hours, nay days, and even weeks, with wounds which could scarcely be otherwise than destructive; and several cases are recorded in which the cicatrices discovered after death, in persons known to have been wounded in the vicinity of the heart, have shown that even severe wounds of that most important organ are not necessarily fatal. As our knowledge of the nature of the injury inflicted can never be distinct, it follows that every wound should be considered as curable until it is unfortunately proved to the contrary.

*Auscultation* and *percussion*, and principally auscultation of the whole præcordial region, have afforded means of judging of injuries of the heart which were not form erly known. A vertical line, coinciding with the left margin of the sternum, has about one third of the heart, consisting of the upper portion of the right ventricle, and the whole of the left, on the left. The apex of the heart beats between the cartilages of the fifth and six left ribs, at a point about two inches below the nipple, and an inch on its external side; or, if one leg of a compass be fixed at a point midway between the junction of the cartilage of the fifth rib on the left side, with the rib and the sternum, and a circle of two inches in diameter be drawn around, it will define as nearly as possible the space of the præcordial region occupied by the heart—whilst uncovered except by the pericardium and some loose cellular texture. In the rest of the præcordial region it is covered and seperated from the walls of the chest by the intervening lung.

If the chest of the dead subject be transfixed with long needles, it will be found that the centre of the first bone of the sternum corresponds with the lower edge of the left subclavian vein, and to the arch of the aorta crossing the trachea; the centre of the second bone to the upper edge of the appendix of the right ventricle; and the centre of the third bone corresponds to the right side of the right auricle; the right ventricle being lower down. A needle penetrating the chest at the costal extremity of the fifth rib, close to the upper edge of its cartilage, will touch the septum of the ventricle. The apex of the heart is an inch and a half below this, and inclined to the left side.

The semilunar valves of the pulmonary artery correspond to a spot a little below the centre of the third bone of the sternum. The aortic valves are a few lines below and behind the pulmonary. The mitral valves are a little lower, and still more deeply seated. The pulmonary artery, after touching the sternum inclines to the left, and is found close to the sternum, between the second and third ribs. The aorta ascends to the first bone, and crosses it to form the arch.

One third of the heart, consisting of the upper part of the right ventricle and of the whole of the right auricle, is beneath the sternum; the remainder of the right, with the left ventricle and auricle, are to the left side of that bone.

On applying the ear to the præcordal region, the patient being in the erect position, two sounds are distinguishable in a healthy heart—one duller and more prolonged, the other clearer and shorter; between these there is scarcely an appreciable interval. The period of repose is sufficiently marked before the first or duller sound returns. Of the time thus occupied, one half is filled up by the first or dull sound; one quarter by the second or sharp sound; one quarter by the pause or period of repose.

Twenty-nine theories have been proposed, each accounting for the sound of the heart. The theory of Dr Billing appears to prevail at present, which supposes that the sounds thus heard "are caused by the valves, which, being membranous, each time they resist the reflux of the blood, are thrown into a state of sudden tension, which produces sound."

The impulse of the heart, so far as it can be felt by the touch, depends much on the position in which the body is placed. In the erect position it is heard between the fifth and sixth ribs. In the recumbent posture the impulse is almost imperceptible. It is, perhaps, more observable when the body is turned on the right side, but decidely more so when it is turned on the left. A clearer sound proceeds from a thin, and a duller sound from a thick heart; a sound of greater extent from a large heart, and a sound of less extent from a small one. A more forcible impulse is given by a thick heart, and one more feeble by a thin one; the impulse is conveyed to a longer distance from a small heart.

From a clearer sound we believe in the probability of an attenuated heart, but we argue its certainty from a clearer sound joined with a weaker impulse. A stronger impulse denotes the probability of an hypertrophied heart, but we argue its certainty from a stronger impulse with a diminished sound.

The terms endocardial and exocardial are used to designate the alterations which take place in the sounds of the heart under disease; endocordial when they occur within the heart, and exocardial when they take place upon its surface. The endocardial murmur of disease, or bellows-sound, takes place of, and is substituted in certain cases for, the first or second, or even for both the healthy or normal sounds. The exocardial murmur of disease is heard with the normal sounds, but confusing and overpowering, sometimes overwhelming them by its rubbing or crumpling noise. The natural sounds exist, although rendered imperceptible by the greater distinctness and nearer approach of the unnatural or unhealthy ones.

The heart apart from the pericardium never moves without a sound; the pericardium apart from the heart never gives out one. Under disease the heart gives out the natural sound, diminished, exaggerated, or modified, or, it may be, totally altered. The sounds given out by a diseased pericardium must always be new (there being no old ones), and are described as rubbing, or to-and fro sounds. The pleura when diseased, being a serous structure, like the inner membrane of the pericardium, gives out less marked, but somewhat similar sounds (the "*frottement*" of the French), in particular stages of the disease.

The alterations in the ordinary sounds constituting the endocardial murmurs of the heart heard under disease, depend principally on the altered state of the endocardium, or membrane lining its cavities; the sounds given off, and called exocardial, on an altered state of the serous membrane of the pericardium, are reflected over the outer surface of the heart. The endocardial or bellows sound, when it accompanies the normal sounds o the heart, may result from any kind of derangemen affecting the internal membrane of that organ, particularl rheumatic inflammation, or from any force which ma compress its cavities; it may depend on the altered qual ity of the blood, from anæmia. It should be presen after excessive hæmorrhages have greatly reduced th powers of the sufferer. When this murmur or sound oc curs after injury in the vicinity of the heart, and is accom panied by fever, it indicatas inflammation of the linin membrane, although no local pain, no palpitations, no irregular movements of the heart should be present.

When a murmur or sound is heard of a different kind, possessing the character of friction, or of surfaces moving backward and forward on each other, or to and fro—this sound is the sign of inflammation of the membrane covering the heart, as well as of that lining the fibrous external tissue of the pericardium. The signs of both external and internal inflammation may be present at the same time, and they frequently are in cases of acute rheumatism.

When the heart is supposed to be wounded, even without much loss of blood, there is fainting, palpitation, irregular movement or total cessation of its action; coldness of the extremities; ghastliness of countenance, succeeded by great anxiety; a sense of anguish; an intermission or cessation of pulse, followed, if the patient should survive, by re-action, which renders it very frequent, and sometimes increases its impulse, whilst the anxiety is increased by pain, sometimes intolerable, referred to the part. These symptoms imply a serious injury, although they may not all be present, and many of them differ in intensity. If the patient should survive, the ordinary sounds of the heart will return, with more or less irregularity, accompanied after a few hours by the endocardial murmur, although something like it may perhaps be observed from the first period of injury. The friction, or attrition sound, indicating the presence of inflammation of the pericardium, may be absent, and will not be discernible, if a layer of blood is effused into the cavity of that membrane, whilst the natural sounds of the heart are rendered more indistinct as the heart is separated from the walls of the chest by the effusion, which distends the pericardium, and impedes the regular action of, but cannot compress the heart, as an empyema does the lung. If inflammation take place without an effusion of blood, the friction sound will be heard, and will usually continue even after some effusion of serum and of lymph have occured, as the quantity of serum is rarely sufficient to prevent the effused and attached portions of lymph from rolling against each other.

The presence of a larger quantity of fluid may be more distinctly known by percussion; if it can be borne in cases of injury, the degree and extent of the dulness being the measure of its existence and accumulation. It may extend over a part or the whole of the præcordial region, reaching as high as the second, or even the first rib, beneath the sternum, and even under the cartilage of the ribs of the right side.

That the heart when wounded is capable of recovery by the permanent closure of the wound, in a few rare instances, is indisputable; and it would seem, from a consideration of the different cases which have been recorded, that such recovery takes place in consequence of there being little blood discharged through the wound, or into the cavity of the pericardium, or into that of the pleura.

The absence or the cessation of hæmorrhage, by the contraction of the wound, or the formation of a coagulum, is the first step towards a cure, and it was to one or other of these circumstances that most of those who survived the injury for several days or weeks owed their existence for the time, although they usually died from the effects of inflammation, more of the inner lining and outer covering, than of the substance of the heart itself.

If the wound be inflicted by a musket or pistol-ball, it cannot be closed, although pressure may be made upon it for a time, so as to suppress the external flow of blood. If this should succeed, it is more than probable that the hæmorrhage will continue internally, and that the patient may die after much suffering, principally from oppression, caused by the escape of blood into the cavity of the chest.

If the wound be a stab, the external opening may be accurately closed, and the escape of blood prevented; but as the pressure of the blood in the pericardium is unequal to restrain the action of the heart, blood forced out through the opening fills the cavity of the pleura, and causes suffocation, unless from some accidental circumstance the opening in the heart becomes obstructed and the bleeding ceases.

If all the circumstances be considered, there can be no doubt of the propriety of closing the wound in the first instance, if the flow of blood is excessive and appears likely to endanger life. It seems to be as little doubtful that the wound should be re-opened after a time, if the danger from suffocation be imminent. The relief obtained by the escape of a little blood may be efficacious, whilst it does not necessarily follow, although it is more than probable it will be so, that its place will be occupied by a further extravasation of blood, which will prove fatal. It is a choice of difficulties, and death from hæmorrhage is easier than death from suffocation.

In the case of the Duke de Berri, whose right ventricle was wounded, and who died from loss of blood, Steifensand reprehends Dupuytren for having opened the external wound every two hours to prevent suffocation; but if death were actually impending from the filling of the cavity of the chest being about to cause suffocation, there was nothing to be done but to give relief at all hazards.

When the sufferer has recovered from the imminent danger attendant on the infliction of the injury, and the pericardium is believed to be so full of blood or of serum as to prevent in a great measure the movements of the heart, it has been proposed by the Baron Larrey to open the pericardium by the following operation—equally, as he thinks, applicable in an ordinary case of hydrops pericardii:—

"An oblique incision is to be made from over the edge of the ensiform cartilage, to the united extremities of the cartilages of the seventh and eighth ribs. The cellular tissue being divided with some fibres of the rectus and external oblique muscles, there remain only a portion of the peritonæum, called its false layer, above the pericardium, which can be seen after the division of all intervening cellular tissue, projecting between the first and second digitations of the diaphragm. Into this the bistoury is to be entered, with the precaution of doing it with the edge turned upward, and directed a little from right to left, to avoid the peritonæum. The smallest portion possible of the anterior border of the diaphragm is next to be divided, where it is attached to the inner part of the cartilage of the seventh rib. The internal mammary artery is to the outside. The patient should be placed perpendicularly, and supported on his bed, which inclines the anterior part and base of the pericardium to the fore part of the chest."

Skeilderup recommends this operation to be done by trepanning first the sternum, a little below the spot where the cartilage of the fifth rib is united to that bone, at which part the periosteum lining it offers considerable resistance, and should not be divided by the trephine. Below this there is a triangular space formed by the separation of the layers of the mediastinum free from cellular tissue, and tending a little more to the left than to the right. The apex of this triangle is opposite the fifth rib; its base touches the diaphragm. The bone having been removed, the patient is made to lean forward, when the projection of the pericardium will enable the operator to feel that a quantity of fluid is within, and to open it with safety.

J. Dierking, a stout muscular man, of the third regiment of German Hussars, was wounded at the battle of Waterloo by a lance, which penetrated the chest between the fifth and sixth ribs, and was withdrawn. He fell from his horse, lost a good deal of blood by the mouth, and some by the wound, and was carried to Brussels without any particular attention being drawn to the injury. His strength not being restored, whilst he suffered from palpitations of the heart, and other uneasy sensations in the chest, he was sent to England to be invalided, and in November, 1815, to York Hospital, Chelsea, in consequence of an attack of pneumonia, of which he died in two days, without attention being particularly drawn to the cicatrix of the wound.

On examining the body I found that the lance, having injured the edge of the cartilage of the rib, passed through the inferior lobe of the left lung, the track being marked by a depressed narrow cicatrix. It then perforated the pericardium under the heart, and sliced a peice of the outer edge of the right ventricle, which being attached below turned over and hung down from the heart to the extent of two inches, when in the fresh state, the part of the ventricle from which it had been sliced being puckered and covered by a serous membrane like the heart itself. The lance then penetrated the central tendon of the diaphragm, making an oval opening, easily admitting the finger, the edges being smooth and well defined. It then entered the liver, on the surface of which there was a small irregular mark or cicatrix. The heart in front was attached to the pericardium by some strong bands, the result of adhesive inflammation, but the general appearance of the serous membrane showed that this had not been either great or extensive. The pericardium was not thickened.

If this man had lived long enough, he might have furnished an instance of hernia of the stomach or intestine into the pericardium.

That the heart when exposed is insensible, or nearly so, to the touch, was known to Galen and to Harvey. Galen is said to have removed a part of the sternum and pericardium, and to have laid his finger on the heart.

Harvey did the same on the son of Lord Montgomery, who was wounded in the chest. Prof. J. K. Jung not only introduced needles into the hearts of animals, but also galvanised them without disadvantage, although Admiral Villeneuve is supposed to have died suddenly from running a pin into his heart with a suicidal intention.

That a person may die from the shock of a blow on the heart, need not be doubted, and that they do die when little blood is lost, is admitted. History preserves the fact, that Latour d'Auvergne, who had obtained the honorable title of "Premier Grenadier de France, and Captain of the 46th demi-brigade," fell and died immediately after receiving a wound from a lance at Neustadt, in the month of July, of the sixth year of the Republic, which struck the left ventricle of the heart, near its apex, but did not penetrate its cavity. He was, however, 68 years of age.

In wounds of the heart, all extraneous matters should be removed, if possible, and all inflammatory symptoms should be subdued by general bleeding, by leeches, by calomel,antimony, opium, &c. The chest should be examined daily by auscultation. If the cavity of the pleura should fill with blood, it ought to be evacuated to give a chance for life, and if the pericardium should become permanently distended by fluid, it should be evacuated.

*Lacerations and ruptures of the heart* have frequently taken place from blows or other serious contusions.

Ollivier, who devoted much time to reading and collecting the observations made by different writers on the injuries of the heart, says, "that of 49 cases of spontaneous rupture of the heart, 34 were of the left ventricle, 8 only of the right, 2 of the left auricle, 3 of the right, and that in 2 cases both ventricles were torn in several places; and that these results were in an inverse proportion to those which occurred after blows or contusions; the right ventricle being ruptured in 8 out of 11 cases, the left ventricle 3 times; the auricles being also torn in 6 out of these 11 cases; the ruptures not being confined to one spot, but taking place occasionally in several different parts, or even in the same ventricle." In 8 of the cases he had noticed, the heart was ruptured in several places. That a spontaneous rupture may be cured as well as a wound, seems likely from a case reported by Rostan, of a woman who died after fourteen years' suffering with pain about the heart, and was found to have the ventricle ruptured. A cicatrix was observed to the left side of the recent rupture, half an inch in extent in every direction, and in which the new matter was evidently different from the natural structure of the heart.—*London Lancet.*

